# Metachronous Squamous Cell Carcinomas of the Uterine Cervix and Lung: True Metachrony or Metastasis

**DOI:** 10.7759/cureus.51922

**Published:** 2024-01-09

**Authors:** Pavel Pavlov, Ivan Galev, Helia Bojilova, George S Stoyanov

**Affiliations:** 1 Clinical Pathology, Complex Oncology Center, Shumen, BGR; 2 Clinical Pathology, Acibadem City Clinic Tokuda University Hospital, Sofia, BGR

**Keywords:** metachronous tumors, human papillomavirus, metastasis, pulmonary carcinoma, cervical carcinoma, squamous cell carcinoma

## Abstract

Human papillomaviruses (HPV) are a big group of infection agents with oncogenic potential, especially regarding squamous epithelium. Some high-risk variants are key in the development of squamous cell carcinomas (SCC) across multiple systems, the most affected of which is the female reproductive system, but also parts of the gastrointestinal tract, head, and neck SCC, and cutaneous and pulmonary (bronchogenic) SCCs. In cases where a patient develops two SCCs in different systems, often the main question is whether these tumors are synchronous, metachronous, or if one of the tumors is a metastasis from the other, with HPV testing and stereotype identification often being of aid in differentiating between these. Herein, we report the case of a female patient in her 50s, initially diagnosed with SCC of the uterine cervix. The patient remained stable for three calendar years after completing preoperative radiotherapy, surgical resection, and postoperative chemo-radiotherapy. At that point, she developed respiratory symptoms, and radiography suggested a pulmonary malignancy. After undergoing surgical resection of the pulmonary lesion, histological specimens were initially interpreted to be a metachronous pulmonary SCC. Immunohistochemical testing proved that both the cervical and pulmonary lesions were HPV-associated, with further testing proving that both lesions were associated with high-risk HPV (genotype 16). Based on the clinical history and aggregated data, the pulmonary lesion was interpreted as a metastatic and not a metachronous one, and the patient is currently undergoing treatment for metastatic disease.

## Introduction

Human papillomavirus (HPV) is a leading culprit for the development of squamous cell malignancies in multiple systems such as the respiratory (upper and lower respiratory tract), gastrointestinal (oral cavity, esophagus, anal canal), skin, and urogenital systems in both genders. In females, HPV infection, especially with highly pathogenic types of the virus, is a dominating mechanism for the development of squamous cell carcinoma (SCC) of the uterine cervix.

Malignancies of the uterine cervix are the fourth most common malignant entry in females [1]. They are dominated by SCC, with only a minority of cases of non-squamous origin and differentiation [2,3]. Of the roughly 80-90% of SCCs in the cervix, nearly 95% of them are HPV-associated [2,4,5]. Based on the patient's presentation and tumor stage, symptoms can vary and include metastatic spread. This often creates a diagnostic problem, rarely seen in other malignancies, as one of the typical locations of metastasis is the lung, an organ where primary SCC is also a common entry. This is rarely seen with other common female malignancies with metastatic spread, as glandular and mesenchymal malignancies vary in morphology and immunophenotype based on their site of origin, whereas SCC has virtually identical morphology and immunophenotype [6]. Herein, we present a case of a female patient with a metachronous uterine cervix and pulmonary SCC.

## Case presentation

**Figure 1:**
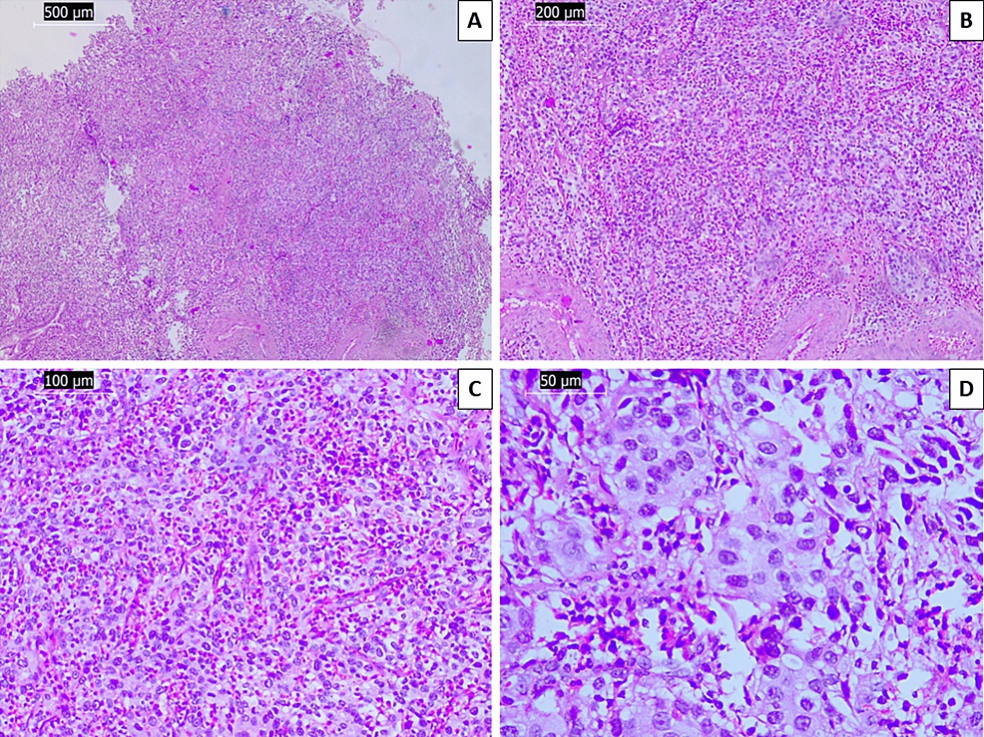
Histopathology of the cervical biopsy A: diffuse proliferation of neoplastic cells, H&E stain, original magnification 40x; B: nested growth pattern of atypical cells, H&E stain, original magnification 100x; C: intervening vascularized stroma, H&E stain, original magnification 200x; D: highly atypical squamous cells, H&E stain, original magnification 400x H&E: hematoxylin and eosin

The patient, a female in her 50s, presented to our institution three years prior due to coital bleeding for the previous three months and a history of atypical squamous cells of undetermined significance on cervical cytology. Previous medical history included a partial thyroidectomy due to goiter and a 7-mm lesion in the right breast with benign characteristics that had remained radiologically and clinically stable for the previous ten years. Colposcopy revealed a 5 mm exophytic lesion on the exocervix, which was biopsied, with histopathology showing a SCC (Figure 1). The patient was scheduled for surgery and received preoperative chemo-radiotherapy as per protocol: six courses of cisplatin and intensity-modulated radiation therapy (IMRT) to a total dose of 45 grays (Gy), fractionated into 1.8 Gy daily doses.

**Figure 2:**
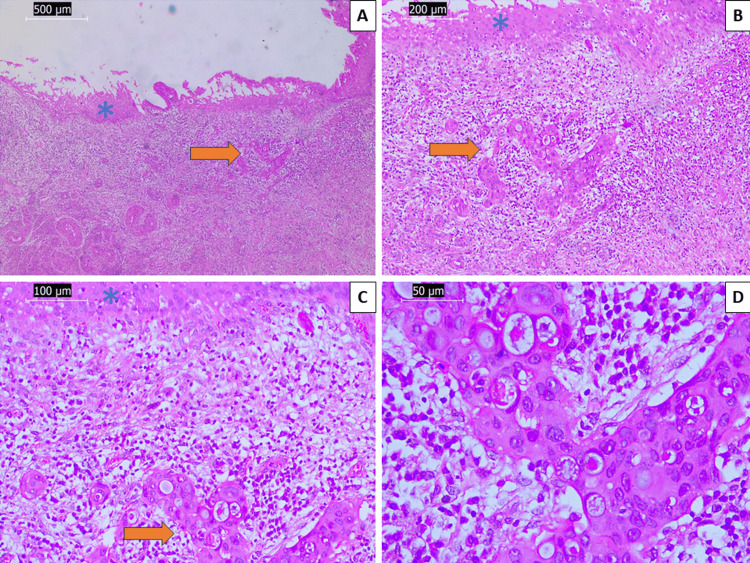
Histopathology of the cervix from the total hysterectomy specimen Dysplastic changes in the superficial epithelium (blue asterisk) and invasive nests (orange arrow), H&E stain, original magnification 40x (A), 100x (B), 200x (C), and 400x showing highly atypical squamous cells within the invasive nests, original magnification 400x (D), H&E stains H&E: hematoxylin and eosin

After histopathology, a decision for total hysterectomy was taken, with histology showing again invasive SCC of the uterine cervix with a depth of invasion of 4 mm, staged as pT1B1 and International Federation of Gynecology and Obstetrics/Fédération Internationale de Gynécologie et d’Obstétrique (FIGO) IB1 (Figure 2). Preoperative computer tomography (CT) showed a heterogenous consistency of the uterine cervix, which had a total size of 61/36/37 mm and no further lesion suspicious for metastasis (on chest and abdomen sections) or invasion (on pelvic sections), and definitive histopathology confirmed the lack of spread outside of the cervix.

After surgery, the patient underwent three courses of postoperative brachytherapy with a total dose of 3.2 Gy each and further photon IMRT to a total dose of 41.4 Gy, fractionated into 1.8 Gy daily doses for prophylaxis toward paraaortic lymph nodes. After completion of radiotherapy, the patient was actively monitored for disease recurrence and progression and remained stable for three calendar years.

On a routine check-up, the patient reported dyspnea and underwent chest radiography, which depicted a cavitating lesion in the left lower lobe with a size of 37/38 mm. Chest CT confirmed the finding as located in the night segment of the left lung, adjacent to the segmental bronchus, which had a thickened mucosa and a lesional size of 45/31/39 mm. Based on the findings, the patient was reevaluated by the oncologic committee and scheduled for a left lower lobectomy.

**Figure 3:**
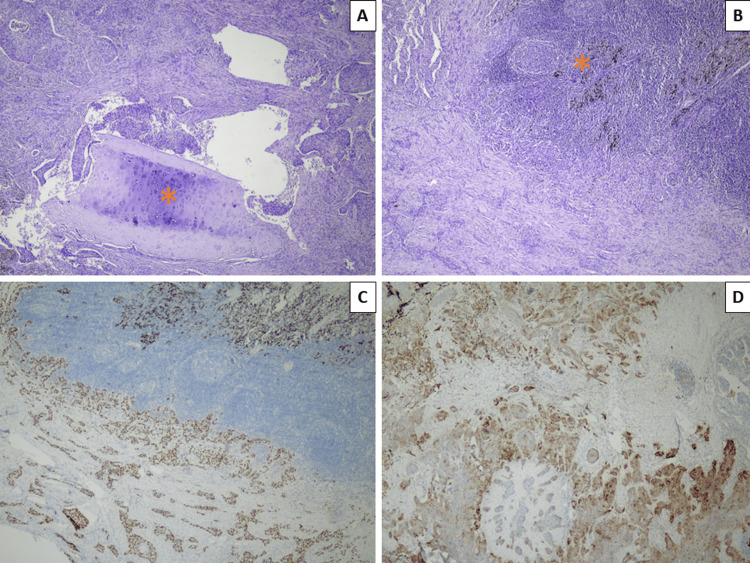
Histopathology and immunophenotype of the resected pulmonary lesion A: nest comprised of highly atypical cells surrounding the cartilage of a large bronchus (asterisk), H&E stain, original magnification x40; B: involvement of a peribronchial lymph node with extensive anthracosis (asterisk), H&E stain, original magnification x40; C: p40 positive stain in tumor cells, original magnification x40; D: PDL1 positive staining in 20% of tumor cells, original magnification x40 H&E: hematoxylin and eosin; p40: Delta Np63 isoform; PDL1: programmed death ligand 1

The patient underwent an uncomplicated robotically-assisted left lower lobectomy. The excised specimen sent for histopathology revealed a tumor with a size of 46/40/30 mm, which did not involve the pleural surface. Resection margins were non-involved. The tumor represented histopathology reminiscent of SCC: a solid nested growth pattern of highly atypical cells with hyperchromatic, dispersed chromatin, and prominent nucleoli. Immunohistochemically, the tumor expressed Delta Np63 isoform (p40) and did not express thyroid transcription factor 1 (TTF1) (Figure 3). The tumor involved both the bronchial wall and an adjacent peribronchial lymph node and showed foci of perineural growth; a further 21 histologically evaluated lymph nodes showed no other metastatic involvement (Figure 3). The tumor was, therefore, initially interpreted to be a lung primary and staged as pT2bN1. The patient was reflectively tested for programmed death ligand 1 (PDL1) expression and epidermal growth factor receptor (EGFR) mutations. EGFR came back as a wild type, while the combined positive score for PDL1 was 20%.

**Figure 4:**
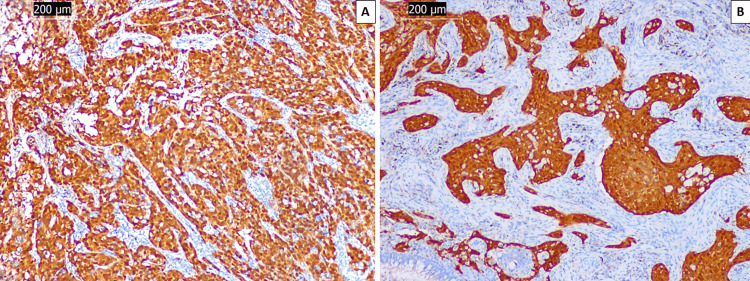
p16 expression in both cervical (A) and pulmonary (B) lesions p16: tumor suppressor protein encoded by cyclin-dependent kinase N2A (CDKN2A)

Despite the histopathology report, the oncologic committee withheld treatment for metachronous pulmonary SCC until further testing to exclude metastasis. Immunohistochemical evaluation of tumor suppressor protein encoded by cyclin-dependent kinase N2A (p16) came back positive in the cervical and pulmonary specimens, confirming the HPV association of both SCCs (Figure 4).

Further multiplexed polymerase chain reaction HPV genotype testing using formalin-fixed block tissues was performed on both lesions for high-risk HPV types, including HPV 16, 18, 31, 33, 35, 39, 45, 51, 52, 56, 58, and 59. Testing on both lesions was positive for HPV 16.

The oncologic committee discussed the patient once again and initiated a treatment protocol for metastatic uterine cervix SCC based on the extensive HPV testing panel. Currently, the patient is undergoing treatment with immune checkpoint inhibitors included and is stable, without new foci of involvement or overall disease progression.

## Discussion

SCCs are common malignant entries across multiple organs and systems [6,7]. Their differential diagnosis, especially in the case of metastatic spread to organs in which SCC can originate, is difficult when compared to metastatic glandular and some mesenchymal entries, in which both morphology and immunohistochemical profile vary significantly more than that of SCCs [6,7]. HPV-associated SCCs are a genotypically specific type of SCC wherein the neoplastic transformation is driven by a transforming HPV infection of the squamous cells [8].

HPV-infected cells, which have the ability to rid themselves of infection, can progress to the so-called transforming infection. In non-transforming infection, viral replication is achieved without viral integration into the host cell genome, in which mitotically active cells have the ability to transmit infection to reproduced cells, wherein it is once again from the non-transforming variety, at least initially [9]. For transforming infection to occur, the viral genome needs to integrate into the host cell genome, which typically occurs in genome-fragile sites. The viral particles produced in such a manner dysregulate cellular proliferation and damage repair mechanisms, basically immortalizing these cells through the inactivation and inhibition of some cyclin-dependent kinase pathways and the retinoblastoma protein. In such cases, when the HPV infection is integrated, i.e., transformative, affected cells overexpress the p16 protein [10]. In this regard, p16 is an excellent immunohistochemical marker for discriminating between HPV-associated and HPV-independent SCC, which will not express p16 on immunohistochemistry [11]. Similar interactions can be observed in some glandular neoplasms once again, predominantly of the uterine cervix; outside of epithelial tumors, p16 expression in mesenchymal tumors, in particular, should be interpreted as intracellular dysregulation independent of HPV infection [12,13].

In our case, the HPV association of cervical SCC is to be considered a borderline classical feature, as HPV-independent ones are rare. Furthermore, the microinvasiveness of the tumor, the complete therapeutic approach, and the late development of the metastatic spread are uncharacteristic. For this reason, the initial clinical and histopathological diagnosis of the pulmonary lesion was that of a metachronous tumor. The histopathological evaluation of the specimens did not reveal lymphonodular metastasis in the majority of the lymph nodes sent for evaluation, apart from one in which the parenchyma was partially involved, the lymphonodular capsule invaded, and a continuous pattern of invasion was noted to the segmental bronchus, and its mucosa was interpreted as a growth pattern of a primary bronchogenic SCC. Unlike the uterine cervix, in which the squamous epithelium is native, bronchogenic (nosologically defined as pulmonary) SCC develops on the basis of a more complex mechanism. As the bronchial tree does not have native squamous epithelium, first squamous cell metaplasia is required to develop on the basis of the chronic toxicity of inflammation. As such, oncogenic mechanisms are typically present within these metaplastic squamous cells without a transforming HPV infection. From a cancer epidemiology standpoint, therefore, even in the presence of HPV infection in other areas of the body, only around 20% of pulmonary SCCs are HPV-associated [14]. Based on the typical process of biological progression of cervical SCC, pulmonary metastasis could be considered a classical feature. Single metastatic foci, especially with nodal and parenchymal involvement and, as in our case, bronchogenic growth, pose a difficult differential diagnosis. The p16 positivity supported the interpretation that both malignant lesions were due to a transforming HPV infection. In such cases, the decision on how to treat the patient is widely up for debate and dependent on the oncologic committee, as the fact that both lesions are HPV positive does not concretely confirm the metastatic nature of the pulmonary lesion in our case. As already stated, up to 20% of bronchogenous SCCs are HPV-associated, with the most common HPV genotype in these lesions being HPV 16 [15,16]. For comparison, while still the most prevalent genotype in cervical SCC, HPV 16 is by no means the most common etiologic HPV serotype leading to intraepithelial neoplasias and SCC of this anatomic location (uterine cervix), with genotype 16 typically thought to account for around 25% and other common high-risk HPV genotypes such as 18, 31, 52, etc. accounting for approximately 10% each [17].

Concrete evidence that would have dramatically changed the course of patient management in our case would have been HPV-associated cervical and HPV-independent SSCs, both being HPV-associated but with different genotypes proven in the lesions. Further testing, which was not performed in our case, which could again have therapeutic implications while still not being absolute in its interpretation, is the sequencing of the viral strands, wherein again, if the viral strains are entirely identical, empirical data would suggest metastasis rather than true metachronal, compared to two viral strands of the same type but with differences in their genotype, which would prove two different strands and hence metachronous lesions [18].

## Conclusions

Several HPV genotypes termed "high risk," such as 16, 18, and 31, are highly oncogenic viruses with significant implications for developing SCCs, but not limited to them, across multiple organs and systems, the most common site being the uterine cervix. As underlined by our case and its management, HPV testing can aid in many aspects when identifying the metastatic spread of SCCs or the presence of synchronous and metachronous SCCs in patients with a tumor finding in more than one system, especially after several years of a stable and non-progressing clinical condition. As in our case, if both lesions are not only HPV-associated but also affected by the same genotype, the interpretation of the case can be debated upon if one of the lesions is HPV-associated and the other is independent, or even if both are HPV-associated but with different genotypes, then this without a doubt proves the individuality of the origin of the tumors.
